# Spironolactone mitigates, but does not reverse, the progression of renal fibrosis in a transgenic hypertensive rat

**DOI:** 10.14814/phy2.14448

**Published:** 2020-05-22

**Authors:** Catherine J. Leader, Darren J. Kelly, Ivan A. Sammut, Gerard T. Wilkins, Robert J. Walker

**Affiliations:** ^1^ Department of Medicine University of Otago Dunedin New Zealand; ^2^ Department of Medicine University of Melbourne Melbourne VIC Australia; ^3^ Department of Pharmacology University of Otago Dunedin New Zealand

**Keywords:** fibrosis, hypertension, kidney, spironolactone

## Abstract

Hypertension plays an important role in the development and progression of chronic kidney disease. Studies to date, with mineralocorticoid receptor antagonists (MRA), have demonstrated varying degrees of results in modifying the development of renal fibrosis. This study aimed to investigate whether treatment with a MRA commenced following the establishment of hypertension, a situation more accurately representing the clinical setting, modified the progression of renal fibrosis. Using male Cyp1a1Ren2 rats (*n* = 28), hypertension was established by addition of 0.167% indole‐3‐carbinol (w/w) to the rat chow, for 2 weeks prior to treatment. Rats were then divided into normotensive, hypertensive (H), or hypertensive with daily oral spironolactone treatment (H + SP) (human equivalent dose 50 mg/day). Physiological data and tissue were collected after 4 and 12 weeks for analysis. After 4 weeks, spironolactone had no demonstrable effect on systolic blood pressure (SBP), proteinuria, or macrophage infiltration in the renal cortex. However, glomerulosclerosis and renal cortical fibrosis were significantly decreased. Following 12 weeks of spironolactone treatment, SBP was lowered (not back to normotensive levels), proteinuria was reduced, and the progression of glomerulosclerosis and renal cortical fibrosis was significantly blunted. This was associated with a significant reduction in macrophage and myofibroblast infiltration, as well as CTGF and pSMAD2 expression. In summary, in a model of established hypertension, spironolactone significantly blunted the progression of renal fibrosis and glomerulosclerosis, and downregulated the renal inflammatory response, which was associated with reduced proteinuria, despite only a partial reduction in systolic blood pressure. This suggests a blood pressure independent effect of MRA on renal fibrosis.

## Graphical abstract

In the setting of severe hypertension, spironolactone reduces kidney fibrosis independent of a hypotensive effect. Mineralocorticoid receptor antagonism reduces hypertensive‐mediated kidney fibrosis.

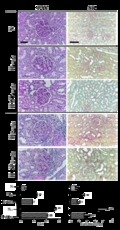

## 1 INTRODUCTION

Hypertension is common in both developed and undeveloped countries (Kearney et al., 2005; Kearney, Whelton, Reynolds, Whelton, & He, 2004), and is considered a major risk factor for global morbidity and mortality (Benjamin et al., 2017; Lim et al., 2012). Sustained, elevated, and untreated hypertension was convincingly demonstrated in the Framingham study (Wolf, Abbott, & Kannel, 1991), to be a strong and consistent predictor of the development of coronary heart disease, transient ischemic attacks, stroke, and congestive heart failure. Hypertension has also been shown to play an significant role in the development and progression of chronic kidney disease (Bidani, Polichnowski, Loutzenhiser, & Griffin, 2013; Bruce, Griffith, & Thorpe, 2015; Hewitson, Holt, & Smith, 2015; Tsioufis et al., 2013).

A number of transgenic hypertensive rat models has been developed to further investigate the pathophysiology of hypertension (Bader, Bohnemeier, Zollmann, Lockley‐Jones, and Ganten 2000; Gomes, Falcão‐Pires, Pires, Brás‐Silva, & Leite‐Moreira, 2013). A recently created model is the transgenic Cyp1a1Ren2 rat (Kantachuvesiri et al., 2001), in which hypertension can be reversibly induced by diet manipulation, to any desired level consistent with survival. In this transgenic rat, mouse Ren2 cDNA expression is under the control of an inducible cytochrome p450‐1a1 promoter, integrated into the Y chromosome of Fischer 344 rats (Kantachuvesiri et al., 2001; Mitchell et al., 2006). Induction of the Cyp1a1 promoter (via dietary indole‐3‐carbinol (I3C)) leads to increased circulating renin levels (Howard & Mitchell, 2012; Kantachuvesiri et al., 2001), activation of the renin–angiotensin–aldosterone system and a consequent increase in blood pressure. Significantly, the degree of hypertension is I3C dose dependent (Mitchell et al., 2006; Peters et al., 2008), allowing for tight titration of blood pressure. Removal of I3C from the diet results in rapid restoration to pretreatment levels of blood pressure (Howard, Mullins, & Mitchell, 2010; Kantachuvesiri et al., 2001; Leader et al., 2018; Peters et al., 2012).

Mineralocorticoid receptor antagonists (MRA), such as spironolactone, have been shown, in animal models and in the clinical setting, to slow the rate of development of cardiac fibrosis and remodeling after cardiac injury (Baldo et al., 2011; Brilla, Matsubara, & Weber, 1993a; Lacolley et al., 2001; Tanaka‐Esposito, Varahan, Jeyaraj, Lu, & Stambler, 2014), which is thought to be both dependent and independent of the actions of angiotensin II (Briet & Schiffrin, 2010; Colussi, Catena, & Sechi, 2012; Messaoudi, Azibani, Delcayre, & Jaisser, 2012; Nagata, 2008). While a number of studies have explored the potential renal protective effects of spironolactone (Barrera‐Chimal et al., 2012; Han et al., 2006; Nielsen et al., 2002; Ortiz, Graciano, Mullins, & Mitchell, 2007), very few studies have been performed in hypertensive animal models and these have reported variable results (Ashek et al., 2012; Baumann et al., 2007; Klanke et al., 2008).

The dose of spironolactone applied in experimental research in rodent models is often well in excess of that used clinically, generally equating using an allometric conversion, to a relative human dose of 550 mg/day or more for an average 70‐kg person (Barrera‐Chimal et al., 2012; Han et al., 2006; Klanke et al., 2008; Taira et al., 2008). Additionally, doses are typically administered at the onset, or prior to onset, of hypertension or kidney insult (Barrera‐Chimal et al., 2012; Fujisawa et al., 2004; Martín‐fernández et al., 2016) and not orally administered. Furthermore, dosing is often for a relatively short time period (less than 2 weeks) (Barrera‐Chimal et al., 2012; Klanke et al., 2008; Ortiz et al., 2007) whereas in humans dosage may be continued for years. Thus, these studies do not reflect the usual clinical setting for hypertension‐mediated injury or when clinical therapeutic intervention occurs.

Therefore, the aim of this study was to investigate whether spironolactone ameliorates renal function and fibrosis over time, following the establishment of hypertension, a situation which more accurately represents the clinical setting.

## 2 METHODS

### 2.1 Animals

The initial transgenic Cyp1a1Ren2 rat (TG [Cyp1a1Ren2]) breeding stock was gifted by Professor J.J. Mullins (Centre for Cardiovascular Science, University of Edinburgh, UK). The transgenic Cyp1a1Ren2 rat colony was held at the University of Otago Animal Resource Unit and animals were housed under controlled conditions of temperature (~21°C) and light (12‐hr light/dark cycle), with food (Meat‐free rat and mouse diet, irradiated, Specialty Feeds) and tap water provided ad libitum. All Cyp1a1Ren2 rats used for experiments were obtained from internal breeding stock and housed in groups of four per cage. All experiments were approved by the Animal Ethics Committee of the University of Otago (AEC #51/13), in accordance with the guidelines of the New Zealand Animal Welfare Act (Ministry for Primary Industries New Zealand, 1999). All rats were gentled by daily handling and weighed before experimental protocols, and weekly thereafter. All animals were humanely euthanized, following final blood pressure measurements (after either 4 or 12 weeks) by halothane inhalation overdose, followed by collection of blood by cardiac puncture, and tissue harvest.

### 2.2 Chronic elevation of blood pressure

**FIGURE 1:**
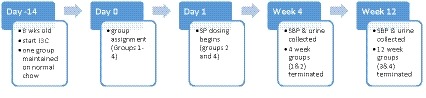
Experimental timeline overview. Hypertension was induced at 8 weeks of age, by addition of indole‐3‐carbinol (I3C) and given 2 weeks to establish before animals were assigned to one of the four experimental groups (Day 0). In addition, one group was maintained on normal chow as a normotensive control. On day 1, daily dosing of spironolactone began in the two treatment groups. After a further 4 weeks, physiological data was collected (urine and systolic blood pressure) from groups 1 and 2 before termination and tissue harvest. The remaining groups (3 and 4) were treated similarly after 12 weeks

Eight‐week‐old male transgenic Cyp1a1Ren2 rats (*n* = 28) were maintained on either irradiated pelleted standard chow (meat‐free rat and mouse diet, Specialty Feeds) to maintain normal blood pressure, or irradiated pelleted standard chow with addition of 0.167% w/w indole‐3‐carbinol (I3C) (#SF13‐086, Specialty Feeds) to induce and maintain elevated blood pressure. Hypertension was established over 2 weeks (day −14 to day 0) (Leader et al., 2018), after which animals were randomized (on day 0) to either a hypertensive group (H) or a hypertensive group administered daily spironolactone (H + SP). Four weeks later, a subset of animals from both group H (*n* = 4) and group H + SP (*n* = 4) were euthanized (by halothane inhalation overdose) for histological examination following blood pressure measurements. The remaining animals (hypertensive (H, *n* = 8), hypertensive plus spironolactone (H + SP, *n* = 4), together with an untreated normotensive group (N, *n* = 8)) were euthanized (by halothane inhalation overdose) following 12 weeks. For these animals, systolic blood pressure (SBP) and weight were recorded after both 4 and 12 weeks. The experimental overview is shown in Figure 1.

### 2.3 Systolic blood pressure

Systolic blood pressure was measured at week 4 and week 12, following establishment of hypertension, in habituated rats, under light sedation with midazolam (1.5 mg/kg, *I.P*.), using tail‐cuff plethysmography (NIBP controller plus PowerLab 4SP, ADInstruments). Animals were given 30 min to acclimatize prior to the blood pressure recording procedure and a heat lamp was used to gently warm the tail prior to SBP readings. Data were captured and analyzed using Chart v.7 software (ADInstruments). Ten clear recordings were taken from each rat on each occasion and the mean value determined.

### 2.4 Spironolactone dosing

Dosing for these animals was calculated using the Food and Drug Administration's 2005 allometric scaling calculations as described by Reagon‐Shaw et al. (2008). To deliver a human equivalent dose of 50 mg/day of spironolactone (Sigma‐Aldrich), 4.41 mg kg day^−1^ was administered to each rat. Experimental animals were dosed orally each day from 10 weeks of age (Day 1), and to enhance acceptance, spironolactone was mixed into a caramel syrup (Quaterpast, Shott Beverages Ltd.).

### 2.5 Urinary protein and creatinine

At 4 and 12 weeks following the establishment of hypertension, rats were weighed and placed into single‐housed metabolic cages (Techniplast), for 12 hr (7 a.m. to 7 p.m.) for urine collection. Urinary Na^+^ and K^+^ concentrations were measured by flame photometry (SEAC SP20) after appropriate dilutions and expressed as a ratio of Na^+^ and K^+^. Urinary protein concentration was measured in triplicate using a BCA assay (Pierce BCA protein assay kit, Thermo Scientific) and expressed as mg/ml, and urine creatinine was measured using a Cobas c310 (Roche) with a creatinine kit (CREJ2 Jaffe Gen.2 kit, Cobas, Roche), expressed as mg/ml, and proteinuria was expressed as a protein/creatinine ratio.

### 2.6 Plasma creatinine

Blood was collected under ethylenediaminetetraacetic acid (EDTA) via cardiac puncture at termination (4 and 12weeks), and centrifuged at 252 *g* for 15min at 4°C. Plasma creatinine was determined in triplicate for each animal using a Randox (CR510) commercial kit.

### 2.7 Quantitative microscopy

A 4–5 mm section was cut transversely from the center of the right kidney, and fixed in 10% neutral buffered formalin (NBF) before being microwaved (55°C, 250 watts) for 5 min. Sections were left in 10% NBF at room temperature overnight before being transferred to 70% ethanol, dehydrated by passage through 90% and 100% ethanol, cleared in xylene and embedded in paraffin wax. Sections were cut at 3 µm and stained with either picrosirius red (SR) with light green counterstain or periodic acid‐Schiff (PAS), followed by mounting in DPX. Stained sections were viewed using a Zeiss Axioplan Microscope (Zeiss), and images of representative regions were recorded using a Nikon microscope camera (DS‐Ri2, Nikon) with Nikon proprietary software (NIS Elements Basic Research imaging software, Nikon).

The extent of renal cortical fibrosis was quantitatively assessed using SR by capturing a minimum of 10 serial, nonoverlapping regions (×50 magnification; an area of 0.34 mm^2^), free of blood vessels, across the mid renal cortex from each animal. The extent of fibrotic tissue was quantified by applying trained pixel classifier software (NIS Elements Basic Research Imaging software, Nikon) to each region and expressed as a percentage of each selected region.

### 2.8 Glomerulosclerosis index (GSI)

For each section, sampling began at a randomly chosen site in the renal cortex of tissue stained with PAS and the section was scanned transversely, examining 50 glomeruli in each section at x100 magnification. The degree of glomerular damage was assessed using a modified semiquantitative scoring method published by Maric, Sandberg, & Hinojosa‐laborde (2004), to give a glomerulosclerosis index (GSI): grade 0, normal glomeruli; grade 1, sclerotic area up to 25%; grade 2, sclerotic area 26%–50%; grade 3, sclerotic area 51%–75%; grade 4, sclerotic area 76%–95%; and grade 5, sclerotic area 95%–100%. The GSI was calculated using the following formula:

$$\text{GSI}=\frac{\left[\left(1\times N1\right)+\left(2\times N2\right)+\left(3\times N3\right)+\left(4\times N4\right)+\left(5\times N5\right)\right]}{\left(N0+N1+N2+N3+N4+N5\right)}$$

where *Nx* is the total number of glomeruli for each grade (Aldigier, Kanjanbuch, Ma, Brown, & Fogo, 2005; Ashek et al., 2012; Bader et al., 2000; Baldo et al., 2011; Barrera‐Chimal et al., 2012) for each animal. Scoring was performed in a double‐blinded manner, and compared using three experienced independent observers.

### 2.9 Immunohistochemistry

Antibodies (diluted in 1% Bovine serum albumin (BSA, Sigma, fraction V)) used were against: connective tissue growth factor (anti‐CTGF, 1:25, polyclonal, sc‐14939, Santa Cruz Biotechnologies), alpha smooth muscle actin (anti‐αSMA, 1:50, monoclonal, A5228, Sigma‐Aldrich), phosphorylated SMAD2 (anti‐phospho‐SMAD2, 1:300, polycolonal, Cell Signalling Technology, kindly donated by Prof D. Kelly), and Cluster of Differentiation 68 (anti‐CD68, 1:100, MA5‐13324, monoclonal, Thermofisher).

Antigen retrieval was carried out (microwave for 10 min in 10 mmol/L citrate buffer at pH 6.0), with the exception of the αSMA antibody, followed by blocking of endogenous peroxidase activity with 3% H_2_O_2_ in PBS. Sections were then preincubated in 1% BSA (Sigma‐Aldrich) in PBS to block nonspecific binding, before labeling with the appropriate antibody overnight at room temperature. Antibodies were visualized using an appropriate horseradish peroxidase‐coupled secondary antibody (anti‐mouse IgG or anti‐goat IgG, 1:25, Dako), followed by incubation with 3,3‐diaminobenzidine substrate (SigmaFast tablets, Sigma‐Aldrich) and counter‐stained with Ehrlich's hematoxylin. After dehydration and clearing, sections were mounted in DPX.

The pSMAD2 staining was performed using an established method (Lekawanvijit et al., 2012). In brief, antigen retrieval was performed in a pressure cooker (4 min at 125°C in Dako target Retrieval solution, pH9, Dako), followed by blocking of endogenous peroxidase activity. Sections were preincubated to block nonspecific binding, before labeling with the anti‐phospho‐SMAD2 antibody overnight at 4°C. Antibodies were visualized using an appropriate horseradish peroxidase‐coupled secondary antibody (Dakocytomation Envison + system labeled polymer (HRP‐linked) anti‐rabbit, Dako), followed by incubation with diaminobenzidine substrate (Dako Liquid DAB + Substrate Chromogen system, Dako) and counter‐stained with Harris's hematoxylin. Sections were dehydrated and cleared before mounting in DPX.

Negative controls were carried out either by omitting the primary antibodies and/or by using appropriate blocking peptides. All analyses were performed in a blinded manner and cross checked by a second, experienced, blind observer.

### 2.10 Quantification of immunohistochemistry

For CTGF, a minimum of 10 nonoverlapping, renal cortical regions (×50 magnification), containing no vessels, were captured for analysis. The degree of CTGF expression was assessed using a semiquantitative scoring method to give a CTGF tubular index score: grade 1, a few tubules showing light or thin and/or interrupted positive (brown) stain; grade 2, the majority of the tubules are positively stained although expression appears mainly adjacent to the basolateral membranes with moderate intensity; grade 3, all tubules are positively stained, expression is intense and not restricted to the membrane; and grade 4, tubules are intensely positively stained with expression throughout the tubular cytoplasm. The CTGF tubular index score was calculated using the same formula to that used for GSI.

For αSMA, a semiquantitative scoring system was used to assess tubulointerstitial fibroblast proliferation in each captured section. The semiquantitative scoring method was: 0—No interstitial fibroblasts, 1—>10 myofibroblasts per field, 2—10–100 myofibroblasts per field, 3—1–5 tubules encased in myofibroblasts, 4—many tubules and glomeruli totally encased by myofibroblasts. Twenty‐five fields (50× magnification) were examined in each section and a mean score was calculated from the total for each section.

For CD68, positively stained interstitial macrophages were counted in each captured image (×100 magnification, 800 ± 10 µm^2^) and calculated as positive stained cells per unit area (mm^2^). A minimum of 10 fields from the renal cortex was captured from each animal section.

Phosphorylated SMAD2 was quantitatively assessed by capturing 20 serial, nonoverlapping regions (×100 magnification), containing no blood vessels, across the mid renal cortex from each animal. The extent of positive stain was quantified by applying trained pixel classifier software (NIS Elements Basic Research Imaging software, Nikon) to each region and expressed as a percentage of each selected region.

### 2.11 Statistics

Data are presented as mean ± *SD*. Additional 95% confidence intervals are also shown where appropriate. Statistical comparisons were accomplished by one‐way analysis of variance (ANOVA) with Bonferroni post hoc analysis (GraphPad Prism 5, version 5.03, GraphPad software Inc.). Correlations were performed as linear regression with Pearson correlation in GraphPad Prism (version 5.03, GraphPad software Inc.). Results were considered to be statistically significant if *p* values were <.05.

## 3 RESULTS

All the animals started at similar weights (274 ± 25 g) and all rats showed a steady weight gain over the 12‐week experimental period. There was no impact of the IC3 or spironolactone on weight gain over this 12‐week period. Normotensive rats maintained a consistent systolic blood pressure (SBP, 80–104 mmHg) over the study duration (Table 1).

**TABLE 1:** Physiological data from repeated measurements of normotensive (N; *n* = 8), hypertensive (H; *n* = 8), and hypertensive rats dosed daily with spironolactone (H + SP; *n* = 4) for 3 months

		*N*	H	H + SP
SBP (mmHg)	Baseline (day 0)	96 ± 12 [92–100]	143 ± 21^***^ [139–148]	141 ± 11^***^ [137–145]
	4 weeks	94 ± 7 [92–96]	172 ± 14^***^ [169–175]	181 ± 23^***^ [172–190]
	12 weeks	91 ± 13 [88–95]	196 ± 21^***^^‡‡‡^ [191–201]	184 ± 15^***^^††^ [179–189]
Urine Na:K ratio	4 weeks	0.41 ± 0.1 [0.31–0.5]	0.68 ± 0.16 ^**^ [0.54–0.81]	0.81 ± 0.03 ^***^ [0.76–0.86]
	12 weeks	0.38 ± 0.1 [0.3–0.46]	0.64 ± 0.16^**^ [0.5–0.77]	0.37 ± 0.07^†^^‡‡‡^ [0.26–0.48]
Urine protein: creatinine ratio	4 weeks	5.4 ± 0.3 [5.2–5.7]	15 ± 2.4^***^ [12.7–17.2]	16.9 ± 3.6^***^ [11.2–22.5]
	12 weeks	5.4 ± 0.3 [5.2–5.6]	20.7 ± 4^***‡^ [17–24.4]	12.6 ± 2^***^ ^†^ [9.5–15.8]
Plasma creatinine (µmol/L)	4 weeks		102 ± 23 [81–123]	78 ± 21 [52–105]
	12 weeks	83 ± 13 [71–95]	173 ± 31^***^ [148–197]	154 ± 18^***^ [135–174]

Note

Within each group, measurements at each time frame were taken from the same individual animals over the 3 months (with the exception of plasma creatinine). Values are shown as mean ± *SD*, with 95% confidence intervals shown in brackets.

One‐way ANOVA with post hoc Bonferroni multiple comparison.

* Indicates significant difference from N. ^*^ *p* < .05, ^**^ *p* < .01, ^***^ *p* < .001.

^†^ Indicates significant difference between H and H + SP. ^†^ *p* < .05, ^††^ *p* < .01, ^†††^ *p* < .001.

^‡^ Indicates significance difference between 4 weeks and 12 weeks. ^‡^ *p* < .05, ^‡‡‡^ *p* < .001.

Hypertensive rats showed a significant and steady increase in SBP in the two weeks prior to the experimental starting point (from 93 ± 10 mmHg to 160 ± 17, *p* < .001 Table 1), as previously reported (Leader et al., 2018). Over the experimental period, SBP continued to rise more slowly, reaching 172 ± 14 mmHg after 4 weeks and 196 ± 21 mmHg after 12 weeks (Table 1). Hypertension was associated with a significant increase (*p* < .01) in urinary sodium: potassium ratios (Na:K) and urine volumes, consistent with a pressure natriuresis. When compared to normotensive urinary Na:K ratio (0.38 ± 0.09), the urinary Na:K ratio in hypertensive animals stayed consistently elevated after both 4 weeks (0.68 ± 0.16, *p* < .01), and 12 weeks (0.6 ± 0.16, *p* < .01; Table 1). Hypertension was also associated with a significant increase in proteinuria over time (Table 1). In the hypertensive animals, plasma creatinine was elevated over the 3 month experimental duration consistent with the development of chronic kidney disease (Table 1).

Hypertensive animals treated with spironolactone initially showed a similar rise in SBP after 4 weeks when compared to the untreated hypertensive group (181 ± 23 vs. 172 ± 14 mmHg, respectively, *p* < .05). However, the progressive rise in SBP over 12 weeks seen in the hypertensive group (196 ± 21 mmHg) was significantly blunted in animals dosed with spironolactone (184 ± 15 mmHg, *p* < .01; Table 1). Early (4 weeks) urinary Na:K ratio was similar to that of the untreated hypertensive animals (0.81 ± 0.03 vs. 0.68 ± 0.16, respectively). Following 12 weeks of treatment with SP, the urinary Na:K ratio was significantly reduced compared to untreated hypertensive animals (0.37 ± 0.07 vs. 0.64 ± 0.16, *p* < .01), and this was not significantly different from the normotensive animals (0.38 ± 0.1; Table 1). A similar pattern was also seen with proteinuria, where, after 4 weeks, hypertensive animals treated with SP demonstrated no difference from untreated hypertensive animals (1.9 ± 0.4 vs. 1.69 ± 0.28, respectively) but by 12 weeks, SP treatments resulted in significant reductions in proteinuria (12.6 ± 2 vs. 20.7 ± 4, respectively, *p* < .05), although these values were still significantly elevated compared to normotensive animals (Table 1).

### 3.1 Cortical fibrosis

**FIGURE 2:**
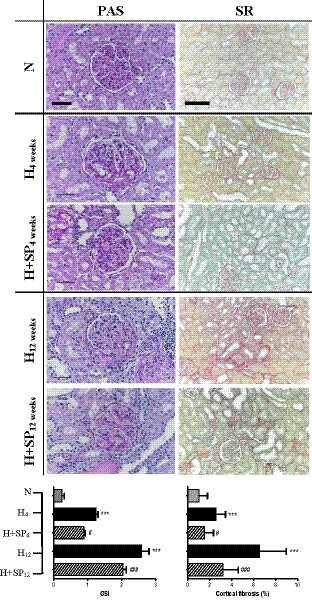
Cortical renal fibrotic changes in hypertensive (H) and hypertensive animals treated with daily oral spironolactone (H + SP) following 4 or 12 weeks compared to normotensive (N) animals. Periodic‐acid Schiff (PAS, scale bar is 50 µm) used to assess glomerulosclerosis. Interstitial cortical fibrosis is shown with picrosirius red (SR, scale bar 100 µm). Below: Histograms show quantification of each stain. Significant difference between N and H is indicated by * (*p < .05, ***p < .001). Significant difference between H and H + SP is indicated by # (^#^ *p* < .05, **p < .01, ***p < .001)

Normotensive animals showed minimal renal cortical fibrosis after 12 weeks (1 ± 0.8%). After 4 weeks, hypertensive animals demonstrated significantly increased cortical fibrosis (2.5 ± 0.9%, *p* < .001) when compared to normotensive animals, predominantly around the distal tubules and cortical collecting ducts. Despite having similarly elevated SBP to the untreated hypertensive group, animals that received spironolactone daily were found to have a significant reduction in renal cortical fibrosis after 4 weeks (1.5 ± 0.8%, *p* < .05), similar to that seen in the normotensive control group (Figure 2).

**FIGURE 3:**
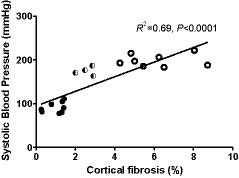
Correlation between systolic blood pressure and renal cortical fibrosis in all groups (normotensive control animals (closed circles), hypertensive animals (open circles), and hypertensive animals dosed with spironolactone (half shaded circles)) after 12 weeks. The *R*‐squared value and significance (*p* value) is shown on the graph

After 12 weeks of untreated hypertension, renal cortical fibrosis had progressively increased (from 2.5 ± 0.9% after 4 weeks to 6.5 ± 2.4% after 12 weeks, *p* < .001). Again, animals treated daily with spironolactone demonstrated significantly less renal cortical fibrosis after 12 weeks compared to the untreated hypertensive animals (3.2 ± 1.4% vs. 6.5 ± 2.4%, respectively, *p* < .001); however, the degree of fibrosis was still greater than that seen in the normotensive controls (*p* < .001; Figure 2). At 12 weeks, the extent of renal cortical fibrosis was significantly correlated with the level of systolic blood pressure across all hypertensive animals (*R* ^2^ = .69, *p* = .0001 – Figure 3).

### 3.2 Glomerulosclerosis

**FIGURE 4:**
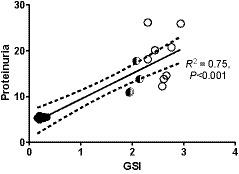
Correlation between glomerulosclerosis (GSI) and proteinuria (expressed as protein: creatinine ratio) in all groups (normotensive control animals (closed circles), hypertensive animals (open circles), and hypertensive animals dosed with spironolactone (half shaded circles)) after 12 weeks. The *R*‐squared value and significance (*p* value) is shown on the graph

Normotensive rats showed minimal fibrotic damage to glomeruli after 12 weeks with a GSI of 0.3 ± 0.06 (Figure 2). In contrast, hypertensive animals had clear evidence of glomerulosclerosis after 4 weeks (GSI of 1.2 ± 0.06, *p* < .001), which was more marked by 12 weeks (GSI of 2.6 ± 0.2, *p* < .001; Figure 2). After 12 weeks, more than 50% of the glomeruli had a GSI score of three or greater (more than 50% sclerotic damage), of which a number of glomeruli were completely fibrosed (5 ± 3.2 per 50 glomeruli assessed; data not shown). Hypertensive animals treated with spironolactone, had significantly less glomerulosclerosis at 4 weeks (GSI 0.89 ± 0.04, *p* < .05) and 12 weeks (GSI 2.03 ± 0.09, *p* < .001), although this was still significant when compared to normotensive animals (Figure 2). Not unexpectedly, the extent of glomerulosclerosis at 12 weeks was significantly correlated with proteinuria across all animals (*R* ^2^ = .75, *p* < .001) (Figure 4).

### 3.3 Fibrosis and inflammation

**FIGURE 5:**
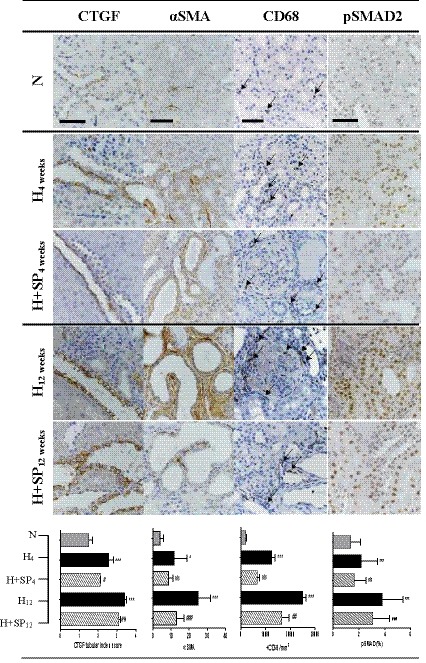
Immunohistochemical renal fibrotic changes in hypertensive (H) and hypertensive animals treated with daily oral spironolactone (H + SP) following 4 or 12 weeks when compared to normotensive (N) animals. Immunohistochemical stains showing expression of connective tissue growth factor (CTGF), alpha smooth muscle actin (αSMA), and phosphorylated SMAD2 (pSMAD2). Macrophage infiltration is shown by positive expression of CD68 (indicated by arrows). Positive expression of each antibody is shown by brown stain. Scale bars 50 µm. Below: Histograms show quantification of each antibody. Significant difference between N and H is indicated by * (*p < .05, ***p < .001). Significant difference between H and H + SP is indicated by ^#^ (^#^ *p* < .05, ^##^ *p* < .01, ^###^ *p* < .001)

Normotensive animals had low levels of renal cortical fibrosis, as assessed by Sirius red staining (Figure 2) and this was associated with low numbers of interstitial macrophages (CD68 positive; 1887 ± 979 macrophages per mm^2^), low levels of αSMA expression (αSMA; 4 ± 1.9%), low levels of pSMAD2 expression (1.4 ± 0.8%), and connective tissue growth factor expression (CTGF; 1.5 ± 0.2%) (Figure 5).

After 4 weeks, hypertensive animals had significantly increased macrophage infiltration (12,441 ± 2,599 macrophage/mm^2^, *p* < .001), increased tubular αSMA expression (11.7 ± 7.2, *p* < .001), increased levels of pSMAD2 expression (2.2 ± 1.3%, *p* < .001), and increased CTGF expression (2.6 ± 0.2, *p* < .001) when compared to normotensive animals. In comparison with the hypertensive animals, animals treated with spironolactone daily for 4 weeks revealed a significant reduction in CTGF expression (2.1 ± 0.03, *p* < .01, Figure 5), but no significant change to macrophage infiltration, αSMA or pSMAD2 expression, despite an overall reduction in cortical fibrosis as shown by Sirius red staining.

Chronic untreated hypertension (12 weeks) resulted in a progressive increase in renal cortical fibrosis (Figure 2) and a significant increase in macrophage peritubular infiltration (24,963 ± 3,335 macrophage/mm^2^ *p* < .01) compared to both the 4‐week untreated hypertensive group and the normotensive animals (12,441 ± 2,599 and 1887 ± 979 macrophages per mm^2^, respectively, Figure 5). Chronic hypertension was also associated with a significant and progressive increases in αSMA expression (peri‐glomerular and tubular) and a significant increase in pSMAD2 (3.8 ± 1.6%) and tubular CTGF expression (3.4 ± 0.1%), when compared to hypertensive animals after 4 weeks (*p* < .001) or to normotensive animals (*p* < .001, Figure 5).

In contrast, long‐term (12 weeks) daily spironolactone administration in hypertensive animals resulted in a significant reduction in macrophage numbers in the renal cortex (16,629 ± 5,863 macrophage/mm^2^, *p* < .01) compared to untreated hypertensive animals at the same time point. Additionally, spironolactone treated animals had reduced CTGF expression (*p* < .05), reduced pSMAD2 expression (*p* < .001), and reduced myofibroblast infiltration (αSMA; *p* < .001), when compared to the untreated hypertensive animals (Figure 5).

Interestingly, although the xpression of αSMA, CTGF, and pSMAD2 was reduced with spironolactone treatment (when compared to untreated hypertensive animals), αSMA, CTGF, pSMAD2 expression was still elevated over time compared to normotensive animals. (*p* < .001). A similar result was seen with macrophage infiltration, as shown by CD68 staining (Figure 5).

## 4 DISCUSSION

Severe hypertension in this Ren2 animal model over 12 weeks, resulting from upregulation of the renin angiotensin aldosterone axis, led to significant hypertensive nephrosclerosis with glomerulosclerosis and renal cortical interstitial fibrosis. This was associated with decreased kidney function and significant proteinuria. There was an intense inflammatory response in the kidneys as evidenced by increased macrophage infiltration in the interstitium, as well as activation of myofibroblasts and profibrotic cytokines such as CTGF and its downstream modulator SMAD2. Aldosterone may contribute to the renal inflammation and injury via modulation of cytokine expression, which promotes inflammatory cell infiltration and albuminuria in hypertension‐driven renal injury (Blasi et al., 2003; Martín‐fernández et al., 2016). In addition, this may be due to upregulation of mineralocorticoid receptor expression in various vascular beds including the renal microcirculation (Delano & Schmid‐Schönbein, 2004), as well as modulating macrophage function (Rickard et al., 2009; Usher et al., 2010).

Commencement of MRA at therapeutically relevant doses after the establishment of hypertension, to mimic clinical intervention scenarios, resulted in a blunting of the hypertension over the 12 weeks to levels similar to that in the untreated hypertensive animals at 4 weeks. More importantly, despite sustained hypertension (184 ± 15 mmHg), over the 12 week period, there was a significant reduction in the progression of hypertension mediated injury with reduced proteinuria, glomerulosclerosis, and interstitial fibrosis. This would suggest both blood pressure dependent and blood pressure independent actions of MRA in slowing the progression of renal fibrosis and glomerulosclerosis.

Ashek and colleagues (2012) using Cyp1a1Ren2 rats (maintained on 0.3% I3C), administered spironolactone (20 mg kg^−1^ day^−1^) for 8 days, demonstrated no effect on hypertension or natriuresis, in agreement with other studies (Klanke et al., 2008; Ortiz et al., 2007), but did demonstrate a reduction in albuminuria. Likewise, we did not see an effect of spironolactone on natriuresis at 4 weeks but this was evident at 12 weeks (Table 1). Ashek and colleagues (2012) attributed this to the marked upregulation of distal tubule expression of renin and pro‐renin receptors with an associated activation of connecting tubule expression of the thiazide sensitive NCC transporter. They also reported a very weak response to amiloride (blocking ENaC) over the same time course. We would therefore speculate that the lack of a natriuresis with spironolactone at 4 weeks may be due to the excessive effects of renin/pro‐renin. However, by 12 weeks of spironolactone therapy, there is sufficient MR blockade to produce a down regulation of ENaC to the extent that enables a natriuresis as evident by the decreased urinary Na/K ratio as we reported. Baumaun and colleagues (Baumann et al., 2007) treated spontaneously hypertensive rats (SHRs) with a dose of 1 mg kg day^−1^ of spironolactone for 4 weeks, and showed a decrease in blood pressure, reduced collagen deposition, and renal tubular hypertrophy, but no attenuation in albuminuria or glomerulosclerosis. Klanke and colleagues (2008) administered a dose of 100 mg kg^−1^ day^−1^ of spironolactone for 2 weeks, following 4 weeks of DOCA‐salt treated Sprague–Dawley rats and reported that spironolactone had no effect on mean arterial blood pressure, but did reduce glomerulosclerosis, macrophage infiltration, and renal interstitial fibrosis. These findings are similar to those we report, albeit with a much higher dose of spironolactone that was used in our model. In a diabetic kidney model, spironolactone reduced aldosterone‐induced CTGF expression and collagen synthesis through a TGF‐β1‐independent manner (Han et al., 2006). In a remnant hypertensive kidney model, MRA inhibition by SP not only slowed development of glomerulosclerosis, but also induced regression of existing glomerulosclerosis in some rats (Aldigier et al., 2005). Studies using the stroke‐prone spontaneously hypertensive rat model demonstrated that spironolactone prevented nephrosclerosis and proteinuria, although the increase in blood pressure was not affected (Rocha, Chander, Khanna, Zuckerman, & Steer, 1998; Rocha, Chander, Zuckerman, & Stier, 1999). A similar effect of spironolactone reducing proteinuria and glomerulosclerosis was described in radiation mediated hypertensive model, again appearing to be a blood pressure independent effect (Brown et al., 2000).

As discussed above, mineralocorticoid receptor antagonists (MRAs) have been demonstrated to attenuate or prevent development of renal failure, renal inflammation and fibrosis, by suppressing the over‐activation of the mineralocorticoid receptor in multiple animal models (Barrera‐Chimal et al., 2012; Du et al., 2009; Fujisawa et al., 2004; Ortiz et al., 2007; Taira et al., 2008). However, in many of published studies, the dose rate utilized, the time when it is administered, the methods of administration, and length of the experimental time does not translate into clinical practice. Most studies using rat models, have used spironolactone doses between 20–200 mg/kg (Barrera‐Chimal et al., 2012; Brilla, Matsubara, & Weber, 1993b; Han et al., 2006; Klanke et al., 2008; Taira et al., 2008). Using the allometric scaling calculations as described by Reagon‐Shaw et al. (2008), a dose of 20–200 mg/kg to a rat is a dose equivalent of 230–2270 mg/day (respectively) for an average 70 kg human. In addition, the route of administration is important to consider. Spironolactone has poor water solubility but with good bioavailability via oral absorption of spironolactone from the GI tract (up to 90%) (Sica, 2005). Therefore the bioavailability and absorbance of spironolactone is likely to be substantially different if administered using non‐oral techniques, and for this reason, only oral and topical formulations have been developed clinically, and other routes of administration, such as intravenous injection (58), are not used. However, most animal studies utilize subcutaneous injections or osmotic pump for delivery, which makes translation to clinical practice more difficult. With limited pharmacokinetic and pharmacodynamic data related to spironolactone in the rat, clearly more detailed pharmacological studies will be required to define spironolactone's action and comparison to equivalent human therapeutic dosing.

There are some limitations related to this study. The number of animals varied from 4 to 8 in the different groups, which some may question. However, given that they are a highly inbred strain, individual genetic variation is likely to be minimal. Significant results were obtained with these small numbers. Also similar studies, using this model has published significant histological data using only 3–5 animals in the different groups (Ashek et al., 2012; Peters et al., 2008). Due to a lack of difference in the physiological data between 1 month and 3 months, only tissue from normal kidneys at 3 months was used as the reference range. It is possible that a small degree of renal fibrosis related to age alone was missed, but given the significant differences between hypertensive and normotensive animals obtained, this would have little or no impact on the results presented. Despite the small numbers, there was a significant difference observed in the extent of fibrosis at 12 weeks between the untreated hypertensive and spironolactone treated hypertensive animals. In this study, two time points were examined, 4 weeks and 12 weeks with or without spironolactone therapy. It would have been interesting to have more time points to examine the potential changes over time.

In this Cyp1a1Ren2 hypertensive model, there is persistent upregulation of renin angiotensin and aldosterone, and clearly there are multiple stimuli producing a profibrotic environment. Angiotensin II is well recognized to have profibrotic cytokine activity (Ruiz‐Ortega et al., 2006) and will have certainly contributed to the changes observed in this study. However, we were interested in identifying the specific effects of MR antagonism in the setting of severe hypertension. In order to examine the complex interaction between angiotensin II and aldosterone and what are blood pressure dependent, as well as independent effects, future studies will include dual blockade with an angiotensin II receptor blocker, as well as mineralocorticoid receptor blockade.

In summary, this study investigated the long‐term impact of clinically relevant doses of spironolactone in the setting of established, chronic, hypertension‐mediated renal injury. This study demonstrated that spironolactone significantly blunted the progression of renal fibrosis and glomerulosclerosis, diminished the renal inflammatory response, and reduced proteinuria in the setting of hypertension‐mediated renal injury, despite no substantial reduction in systolic blood pressure. As aldosterone continues to show a pivotal role in the pathophysiology of nephrosclerosis and renal fibrosis, it further underscores the potential therapeutic benefits of selective aldosterone blockade for treatment of hypertensive renal disease. Further work is planned to categorize the renal dysfunction in this animal model and the potential mechanisms that may mediate the actions of spironolactone.

## CONFLICT OF INTEREST

All the authors have no conflicts of Interest with respect to this publication.

## AUTHOR CONTRIBUTIONS

CL performed the animal work, histology, immunohistochemistry, analysis, and the initial draft of the paper. DK assisted with the immunohistochemistry. RW, IS, and GW contributed to the analysis and interpretation of the data, and were responsible for the initial design and concept of the experiment. All authors contributed to the preparation and review of the final manuscript.
